# Supplementary material for: State-of-the-art global models underestimate impacts from climate extremes
Source: Nat Commun. 2019 Mar 1;10:1005. doi: 10.1038/s41467-019-08745-6 (PMC6397256; doi:10.1038/s41467-019-08745-6)
Supplement: Supplementary file 1 — Supplementary Information [file 41467_2019_8745_MOESM1_ESM.pdf]

Supplementary Material for “State-of-the-art global models underestimate impacts from climate extremes” by Schewe et al.

Contents:

Climate forcing data: Supplementary Figures 1 –3 2

Model ensemble: Supplementary Table 1 4

Water resources: Supplementary Figures 4 –7 5

Agriculture: Supplementary Figures 8 – 9, Supplementary Table 2 9

Terrestrial Ecosystems: Data caveats, Supplementary Figures 10 – 13 12

Energy: Wind and solar power, Supplementary Figure 14 16

Human health: Model description, Supplementary Table 3 16

Marine Ecosystems: Supplementary Figures 15 – 19 17

Methods: Shifted crop yield series, Supplementary Figures 20 – 22 21

References 23

Climate forcing data: Supplementary Figures 1 –3

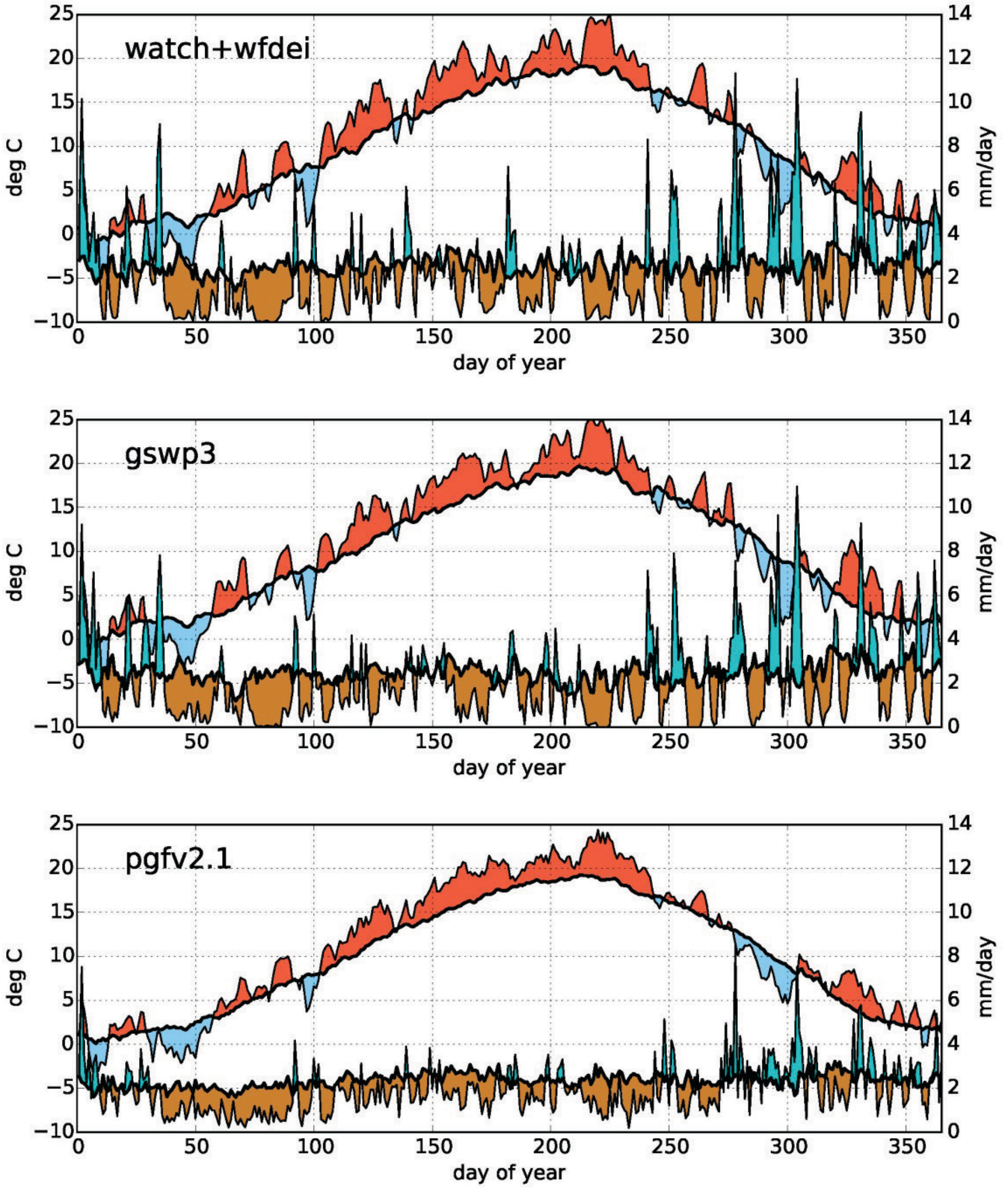

Supplementary Figure 1 Daily temperature (in °C, red/blue shading) and precipitation (in mm/day, turquoise/brown) averaged over the "Central" European region (0°-20°E, 42°-53°N). Thick lines show the 1961-1990 climatology, thin lines show the 2003 values. Red and brown shading means that 2003 was hotter and drier than average, respectively, on a given day of year. In the top panel, the WFDEI data set has been extended by the WATCH forcing data set (1) for the period 1961-1978. WATCH uses the same methodology as WFDEI (2) but is based on ERA-40, rather than ERA-Interim, reanalysis data.

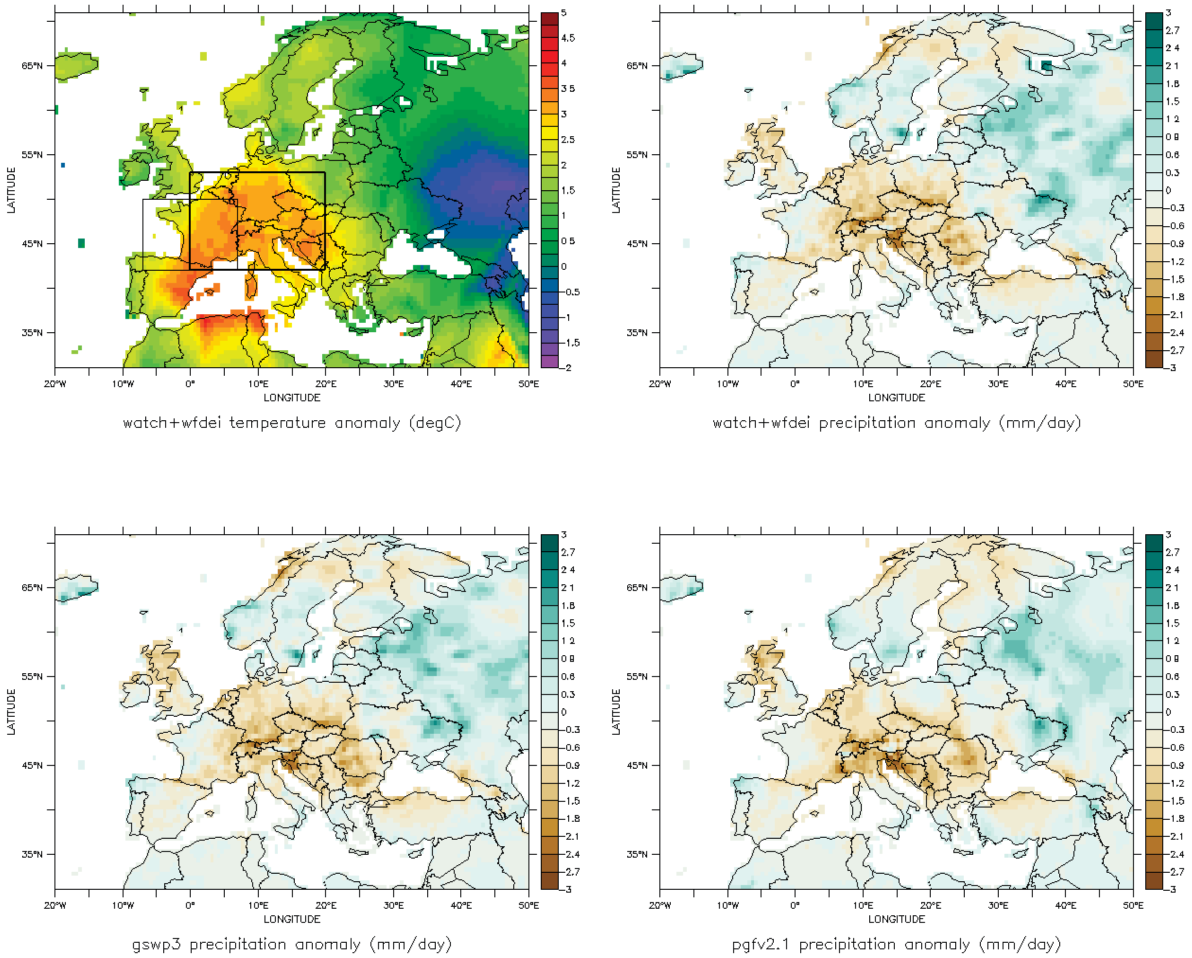

Supplementary Figure 2 Summer (June–August average) climate anomalies in 2003 (with respect to the 1961-1990 average) in the three climate forcing data sets. Top left: Surface air temperature (in °C); only shown for WATCH/WFDEI (2) since monthly temperatures have been bias-corrected using Climate Research Unit (CRU) observational data in all three data sets, and thus are identical. Thick and thin box indicate the “Central” and “West” regions, respectively, used for averaging in the main paper and in Supplementary Figures 1, 10, and 22. Top right to bottom: Precipitation (in mm/day) in WATCH/WFDEI, GSWP3 (3), and PGFv2 (4).

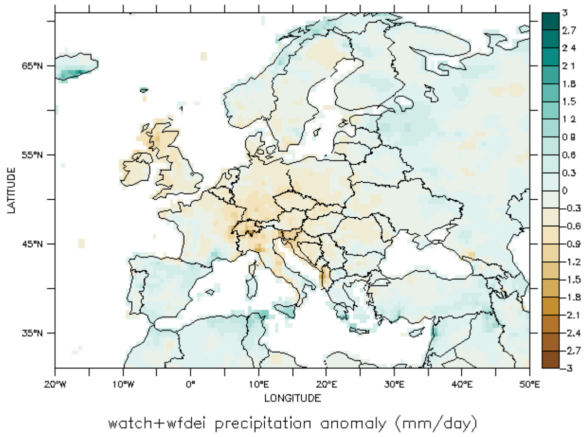

Supplementary Figure 3 As Supplementary Figure **2** (top right) but for the annual average.

Model ensemble: Supplementary Table 1

Supplementary Table 1 Impact models used in this study, including main references

# (as in figures)	Model name (Model version)	Main reference(s)	Code availability
	**Agriculture**		
1	CGMS-WOFOST (WOFOST 7.1/PCSE 5.1)	(5, 6)	https://github.com/ajwdewit/pcse, https://github.com/ajwdewit/ggcmi
2	CLM-Crop (modified CLM 4.5)	(7)	http://www.cesm.ucar.edu/models/cesm1.2/clm/
3	EPIC-Boku (EPIC0810)	(8)	EPICv0810 field-scale core model: https://epicapex.tamu.edu/epic/. Extensions available from model developers upon request
4	EPIC-IIASA (EPIC0810)	(9)	
5	GEPIC (EPIC0810; partly modified at EAWAG)	(10, 11)	
6	LPJ-GUESS (Version 2.1 with crop module)	(12)	upon request
7	LPJmL ()	(13)	Most recent version: https://github.com/PIK-LPJmL/LPJmL. Version used here available upon request
8	ORCHIDEE-CROP (V1.1)	(14)	upon request
9	pAPSIM1.0 (APSIM V7.5)	(15)	https://github.com/RDCEP/psims
10	pDSSAT2.0 (DSSAT4.6)	(15)	
11	PEGASUS (V1.1)	(16)	upon request
12	PRYSBI2	(17)	upon request
	**Terrestrial Ecosystems**		
1	CARAIB ()	(18)	upon request
2	DLEM (Dynamic Land Ecosystem Model) (v2.0)	(19, 20)	upon request
3	JULES-B1 (JULES v4.4)	(21)	https://jules.jchmr.org/
4	LPJ-GUESS (3.1)	(22)	upon request
5	LPJmL ()	(13, 23)	Most recent version: https://github.com/PIK-LPJmL/LPJmL. Version used here available upon request
6	ORCHIDEE (rev3013)	(24, 25)	upon request
7	VEGAS (v2.3)	(26)	upon request for collaboration
8	VISIT (VISITa)	(27)	upon request
	**Water**		
1	DBH ()	(28)	http://hydro.iis.u-tokyo.ac.jp/DBH/
2	H08 (H08)	(29)	http://h08.nies.go.jp
3	LPJmL ()	(30)	Most recent version: https://github.com/PIK-LPJmL/LPJmL. Version used here available upon request
4	MATSIRO (HiGW-MAT)	(31)	not available
5	MPI-HM (R44)	(32)	upon request
6	PCR-GLOBWB (version 2)	(33)	github link at https://www.geosci-model-dev.net/11/2429/2018/
7	WaterGAP2 (WaterGAP 2.2 (ISIMIP2a))	(34)	not available
	**Marine Ecosystems**		
	BOATS (v1.0)	(35)	https://doi.org/10.5281/zenodo.27700

	EcoOcean ()	(36)	EwE core model: http://ecopath.org
	Macroecological model (v1.0)	(37)	upon request
	EwE	(36, 38–40)	http://ecopath.org
	**Energy**		
	VIC-HydroP	(41)	upon request
	**Heat-related mortality**		
	City-specific ERFs	(42, 43)	upon request

Water resources: Supplementary Figures 4 –7

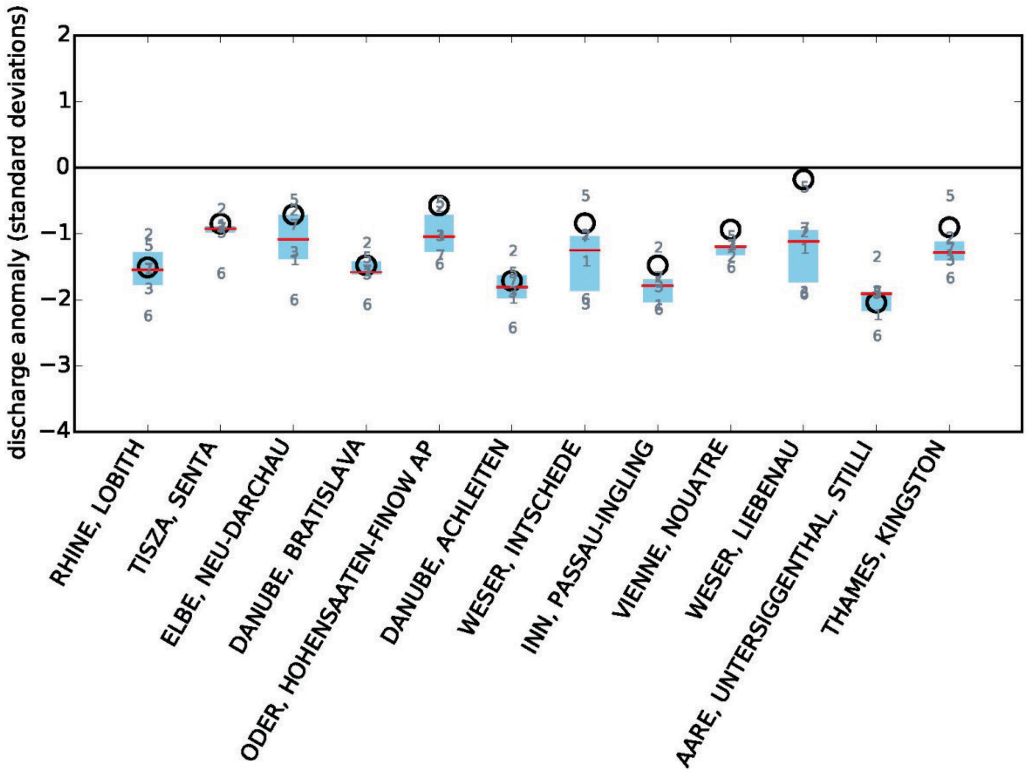

Supplementary Figure 4 As Fig. 2 in the main paper but for simulations ignoring human interventions with the water cycle, such as land use, reservoirs, and water withdrawals. Six models are included here, rather than five for the simulations including human interventions.

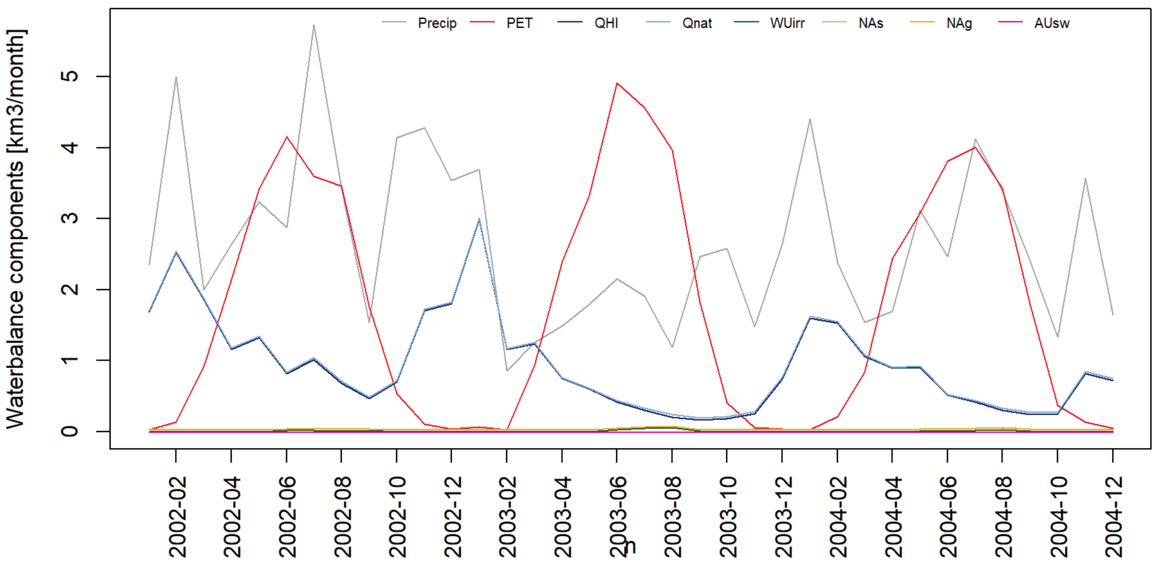

Supplementary Figure 5 Water balance components in the WaterGAP model, driven by WFDEI climate forcing, in the Weser catchment (station Intschede) during three years centered on 2003. Precipitation (Precip), potential evapotranspiration (PET), and discharge (QHI: simulation including human interventions; Qnat: naturalized simulation) range in the order of several km^3/month, while the human water use components are much smaller and cluster at the bottom of the figure (WUirr: consumptive irrigation water demand; NAs: net consumptive demand from surface water sources; Nag: net consumptive demand from groundwater resources; AUsw: actual water consumption from surface water). While irrigation demand is larger in 2003 than in 2002 and 2004 (small bulge at the bottom center), actual water consumption remains near zero (straight purple line) due to a lack of sufficient surface water resources to satisfy the demand. The difference between QHI and Qnat is therefore small.

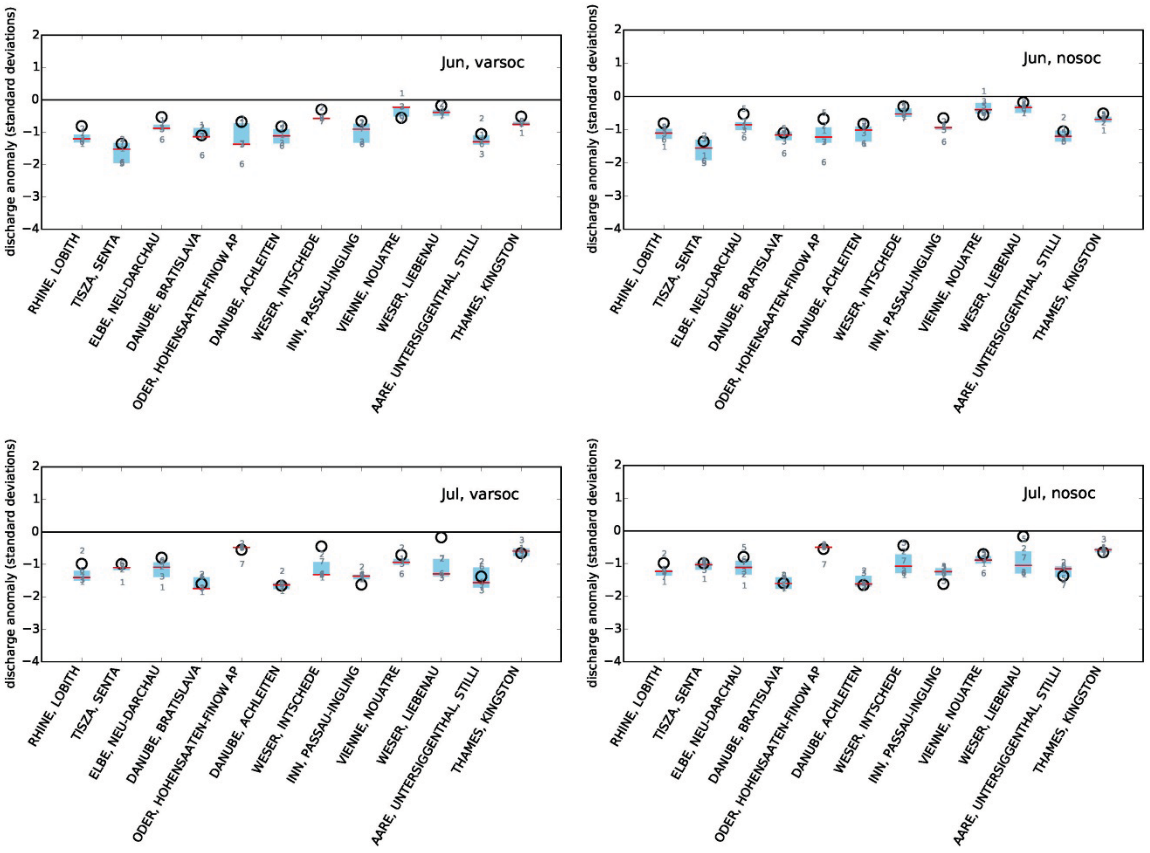

Supplementary Figure 6 As Fig. 2 in main paper (left) and Supplementary Figure **4** (right) but for June and July. The "varsoc" (time-varying societal impacts) scenario includes human interventions, while "nosoc" (no societal impacts) refers to the simulations without human interventions.

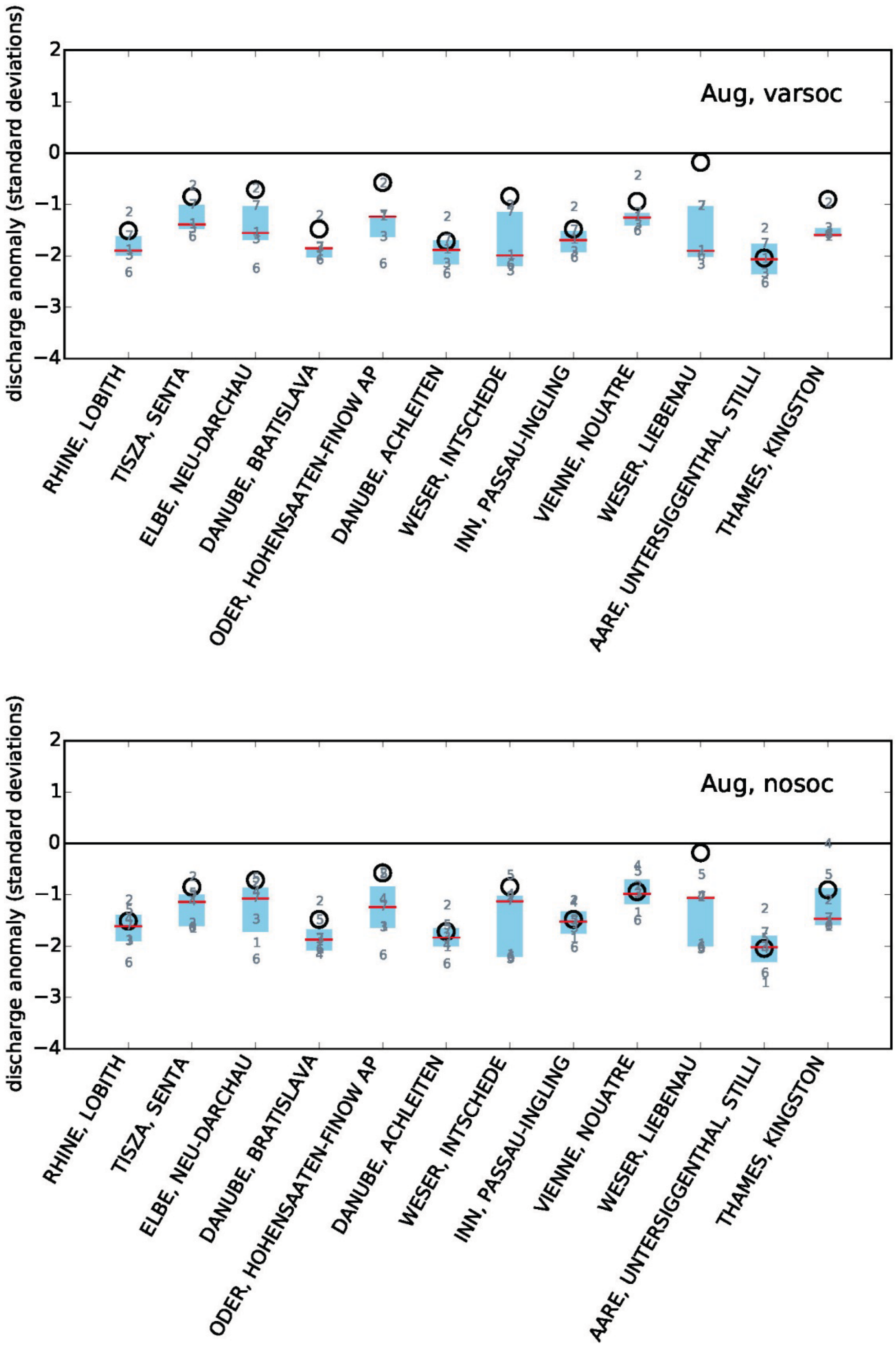

Supplementary Figure 7 As Fig. 2 in the main paper (top) and Supplementary Figure **4** (bottom), but with climate forcing from the GSWP3 dataset. The model ensemble includes 5 models for "varsoc", and 7 models for "nosoc"; individual models are identified by the same numbers as in the main paper.

Agriculture: Supplementary Figures 8 – 9, Supplementary Table 2

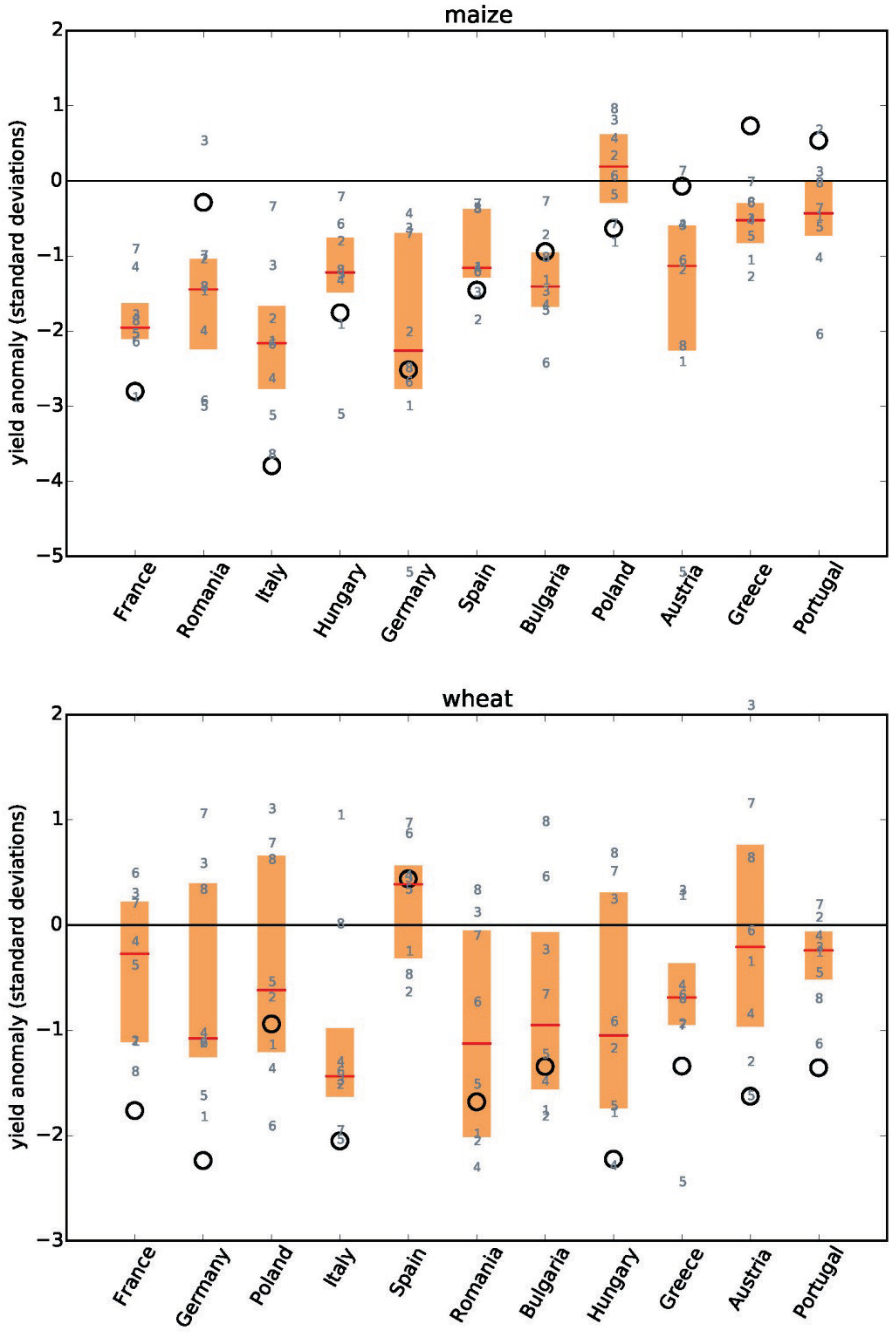

Supplementary Figure 8 As Fig. 3 in the main paper but with PGFv2 climate forcing.

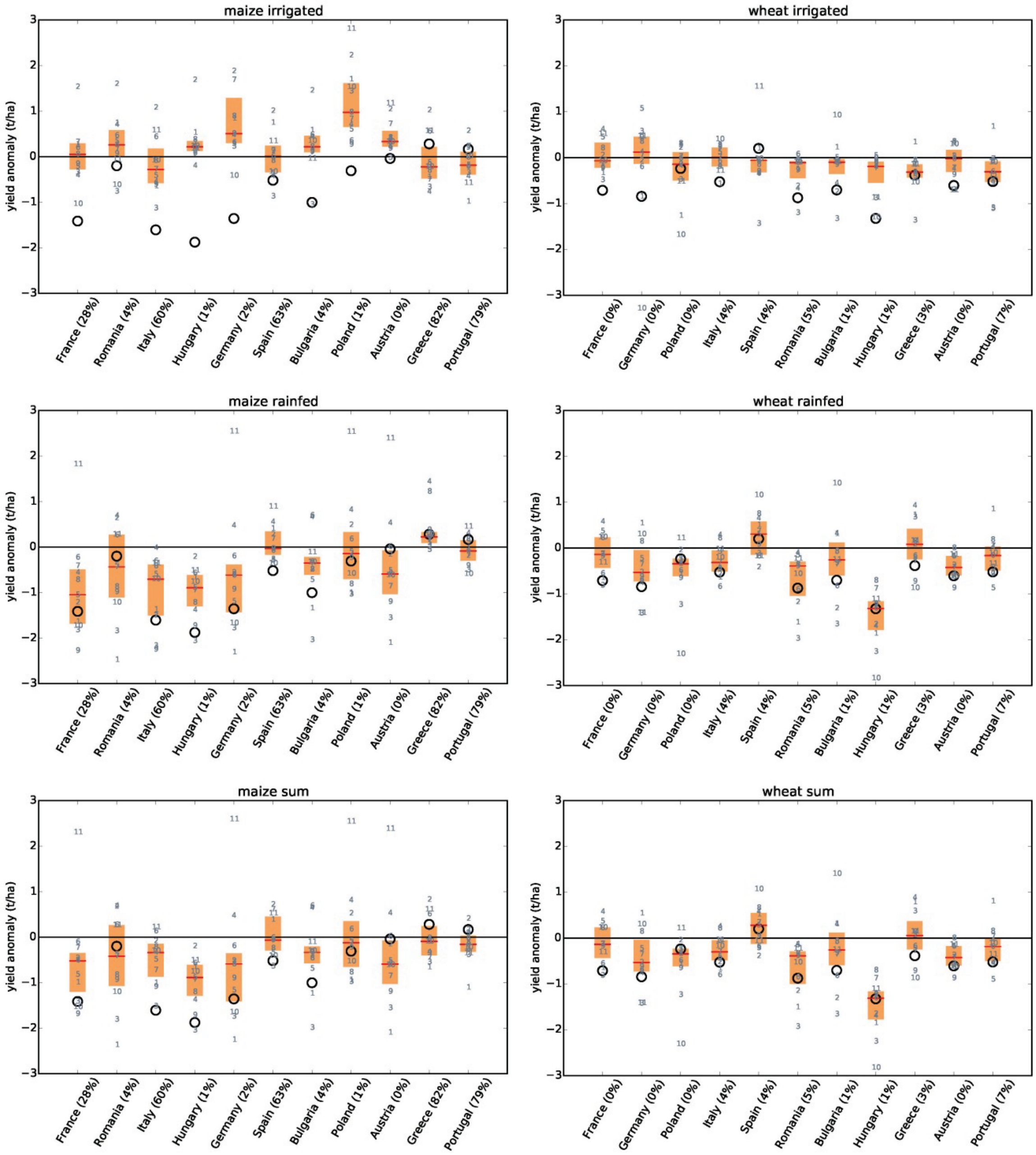

Supplementary Figure 9 As Fig. 3 in the main paper, but in absolute terms (t/ha), and separately for irrigated (top) and rainfed (middle) yields, as well as total yields (bottom). Only 11 crop models are included here because one model (no. 12) does not separate between irrigated and rainfed yields. The percentage of total growing area equipped for irrigation is listed in brackets.

Supplementary Table 2 Comparison of yield changes reported by FAOSTAT (obtained from http://faostat.fao.org/site/567/default.aspx on August 30th, 2016) and COPA-COGECA (44) for those countries which are mentioned in the COPA-COGECA report. COPA-COGECA only reports percentage changes between 2002 and 2003 annual yields, without comparison to longer-term yields.

	**Maize** *% change from 2002 to 2003*		**Wheat** *% change from 2002 to 2003*	
	FAOSTAT	COPA-COGECA	FAOSTAT	COPA-COGECA
Austria	1.8	-9.7	-11.8	-10.4
France	-20.7	-20.0	-16.1	-16.1
Germany	-21.2	-23.0	-5.9	-5.9
Greece			-14.3	-12.6

Italy	-21.2	-26.0	-12.0	-4.7
Portugal			-52.1	-37.0
Spain	-5.0	-13.3		

Terrestrial Ecosystems: Supplementary Figures 10 – 13

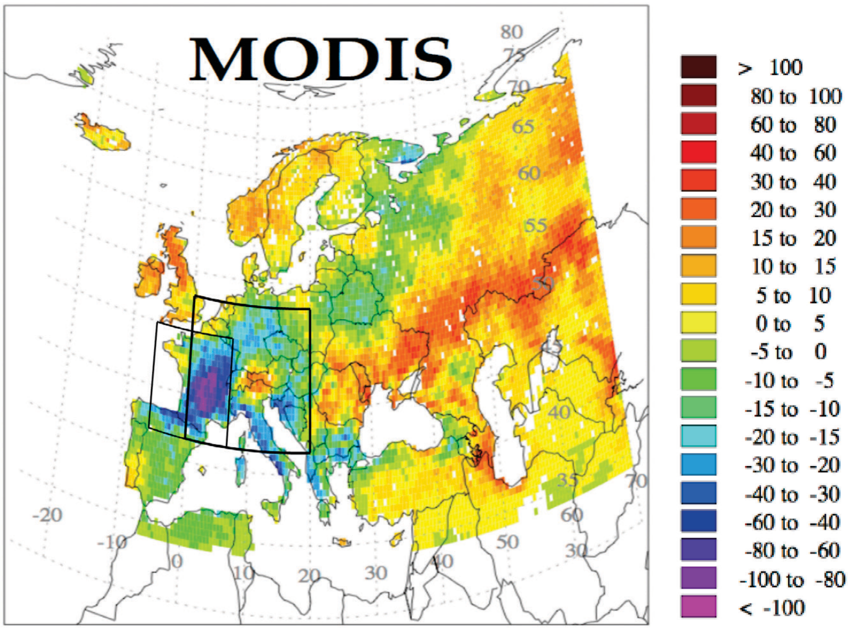

Supplementary Figure 10 Summer (June-August) anomalies in gross primary production (GPP, in gC m^{-2} $month^{-2}$) in the year 2003, relative to the average over 2000-2011, according to MODIS remote-sensing based estimates. Thick and thin black rectangles outline the “Central” and “West” regions, respectively, used for averaging.

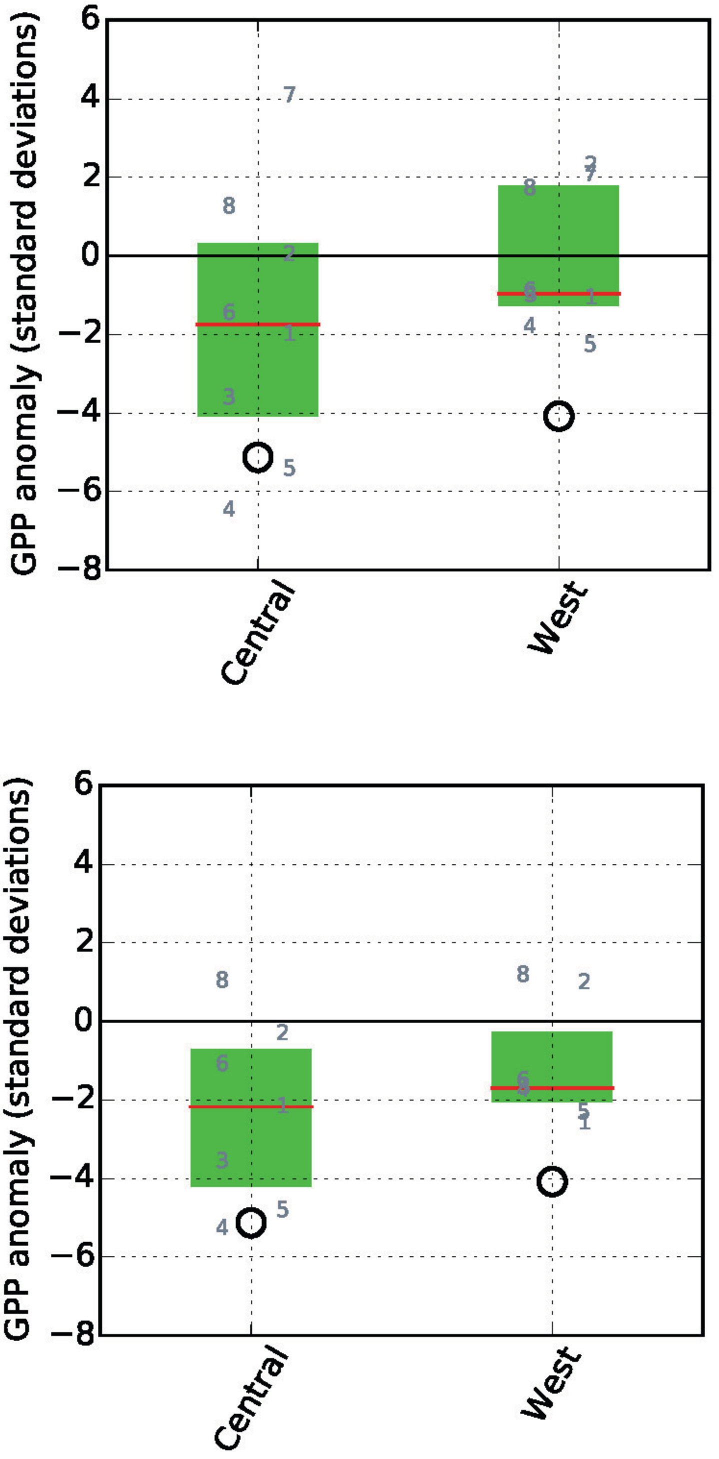

Supplementary Figure 11 As Fig. 4 (a) in main paper but using GSWP3 (top) and PGFv2 (bottom) climate forcing, with simulations starting in 1971.

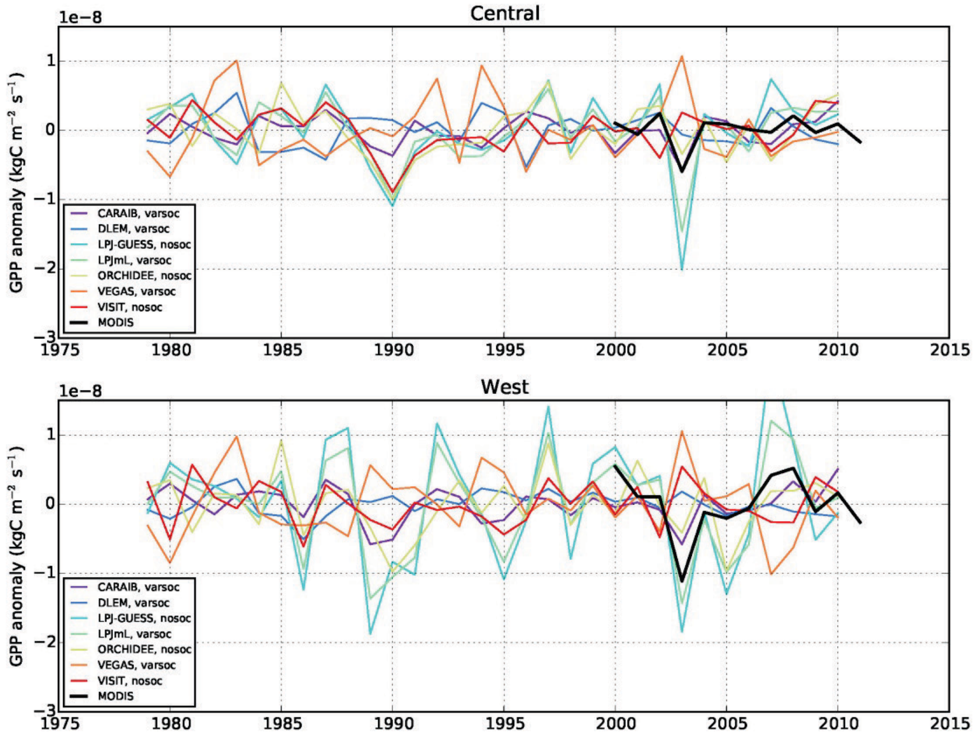

Supplementary Figure 12 Anomalies in summer (June-August) total GPP over the two European regions (see **Supplementary Figure 10**), relative to the linear trend over the entire period (1979-2010 for the biomes models, 2000-2011 for MODIS data), for simulations using WFDEI climate forcing. Models are shown in color, MODIS data in black. Some model simulations (“varsoc”) include time-varying human interventions – most relevant, land-use change – while others (“nosoc”) don’t.

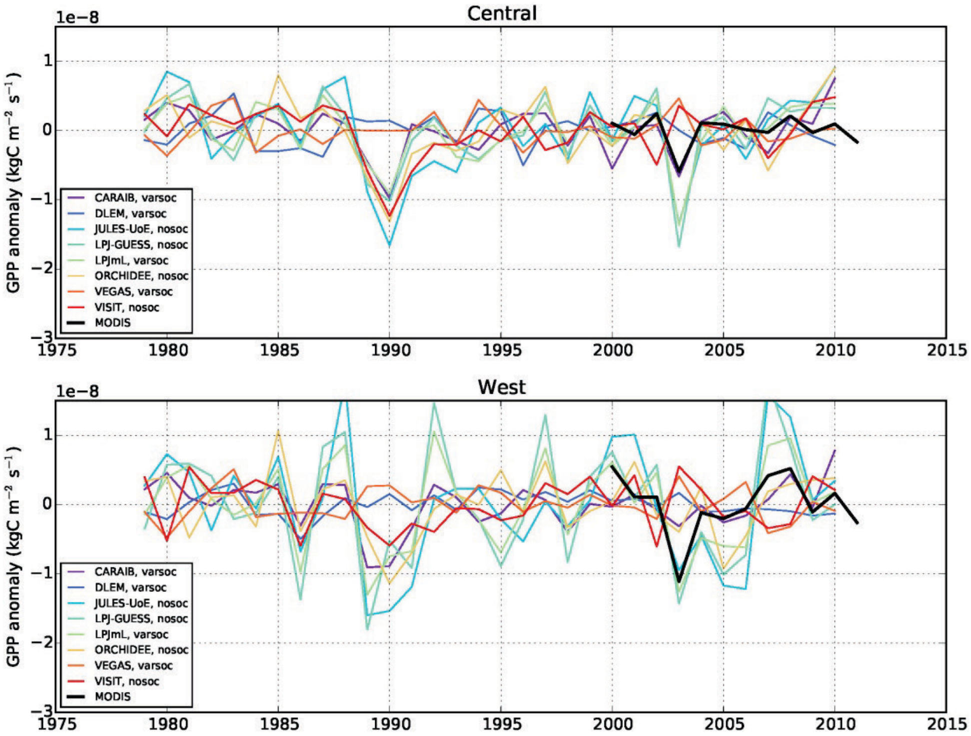

Supplementary Figure 13 As Supplementary Figure **12** but for simulations using GSWP3 climate forcing.

Energy: Wind and solar power, Supplementary Figure 14

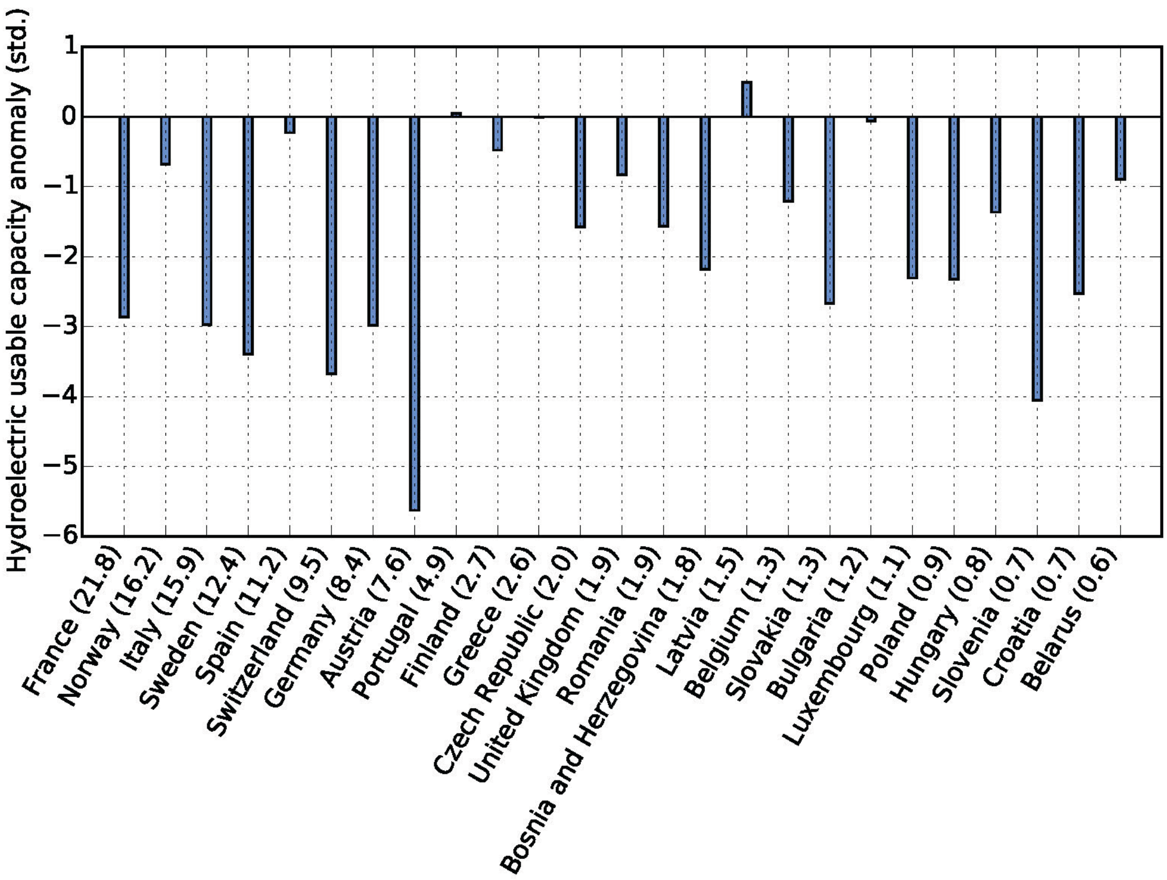

Supplementary Figure 14 As Fig. 5 (blue bars) in main paper but for summer (June-August).

Human health: Supplementary Table 3

Supplementary Table 3 Previous estimates of excess mortality due to the 2003 EHWD in different European cities. Reported numbers of excess deaths are displayed in Fig. 6 (main paper), after normalization by their respective population baseline. In cases where the population baseline is not directly reported in the cited studies, we use official population statistics for 2003 (URLs given in the table), and display the resulting mortality rate as a diamond symbol in Fig. 6 (main paper).

Study	City	Number excess deaths	City population baseline	Time period 2003	Baseline
Michelozzi et al. 2005 (52)	Rome[1]	944	2 546 804 *	Jun-Aug	Smoothed daily mortality 1995-2002
	Turin[1]	577	865 263 *		1998-2002
	Milan[1]	559	1 256 211 *		1995-2002
Le Tertre et al. 2006 (57)	Paris	2085	6,164,418 (baseline 1996-2003)	22 Jul to 2 Sep	Poisson regression models – seasonal predictions as

[1] city population baseline reported in Michelozzi et al., 2006

					baseline
Johnson et al. 2005 (58)	London	616	7,394,817 (https://data.london.gov.uk/dataset/office-national-statistics-ons-population-estimates-borough, 08.08.2017)	4-13 Aug	1998-2002 average
Mitchell et al. 2016 (49)	Paris (Central)	723	2 126 000	Jun-Aug	Baccini et al. models but observed mortality and weather from 2003
	London	322	7 154 000	Jun-Aug	
Borrell et al. 2006 (51)	Barcelona	411	1,582,738	Jun-Aug	Age-group specific model 1998-2002
Tobias et al. 2010 (56)	Barcelona	537	1,582,738 *(http://www.ine.es/dynt3/inebase/en/index.htm?padre=527, 08.08.2017)*	Jun-Aug	Poisson regression 1999-2003
Martinez-Navarro et al. 2004 (55)	Barcelona	665	1,582,738 *(http://www.ine.es/dynt3/inebase/en/index.htm?padre=527, 08.08.2017)*	Jun-Aug	Poisson regression 1990-2002 (account for age groups, month, year)
	Valencia	244	780,653 *(http://www.ine.es/dynt3/inebase/en/index.htm?padre=527, 08.08.2017)*		
Grize et al. 2005 (54)	Zürich	47	342 116 (https://www.bfs.admin.ch/bfs/de/home/statistiken/bevoelkerung.html, 08.08.2017)	Jun-Aug	Poisson regression 1990-2002

* as reported in ref. (53)

Marine Ecosystems: Supplementary Figures 15 – 19

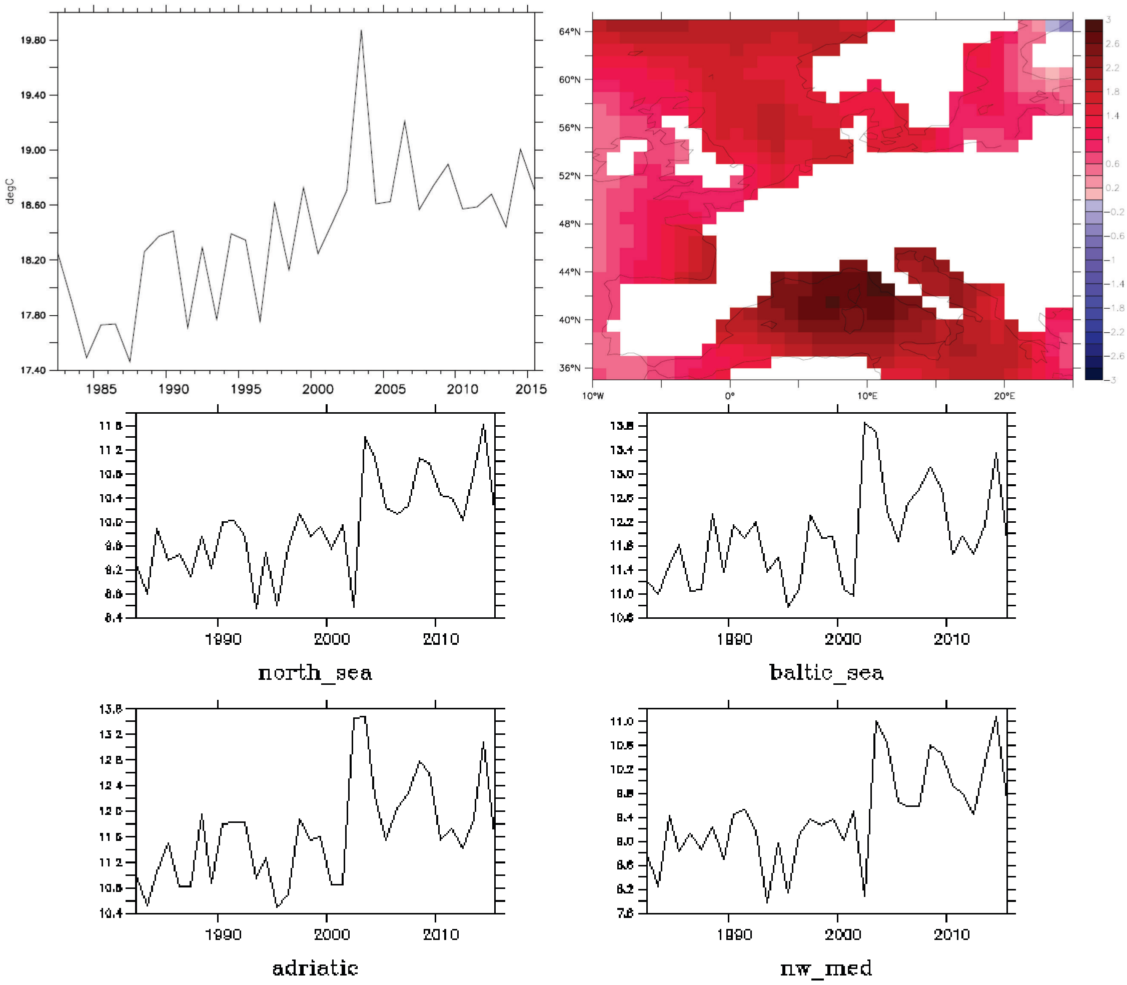

Supplementary Figure 15 Effect of the 2003 heat wave on European sea surface temperatures (SST): Timeseries (top left, average over the region shown on the right) and difference between 2003 and the 1982-2015 average (right, in degree C), both for the summer months June-August (JJA). Bottom four panels show the individual timeseries for the four sub-basins analyzed in the following figures. Source: Observational data from NOAA (http://www.esrl.noaa.gov/psd/data/gridded/data.noaa.oisst.v2.html).

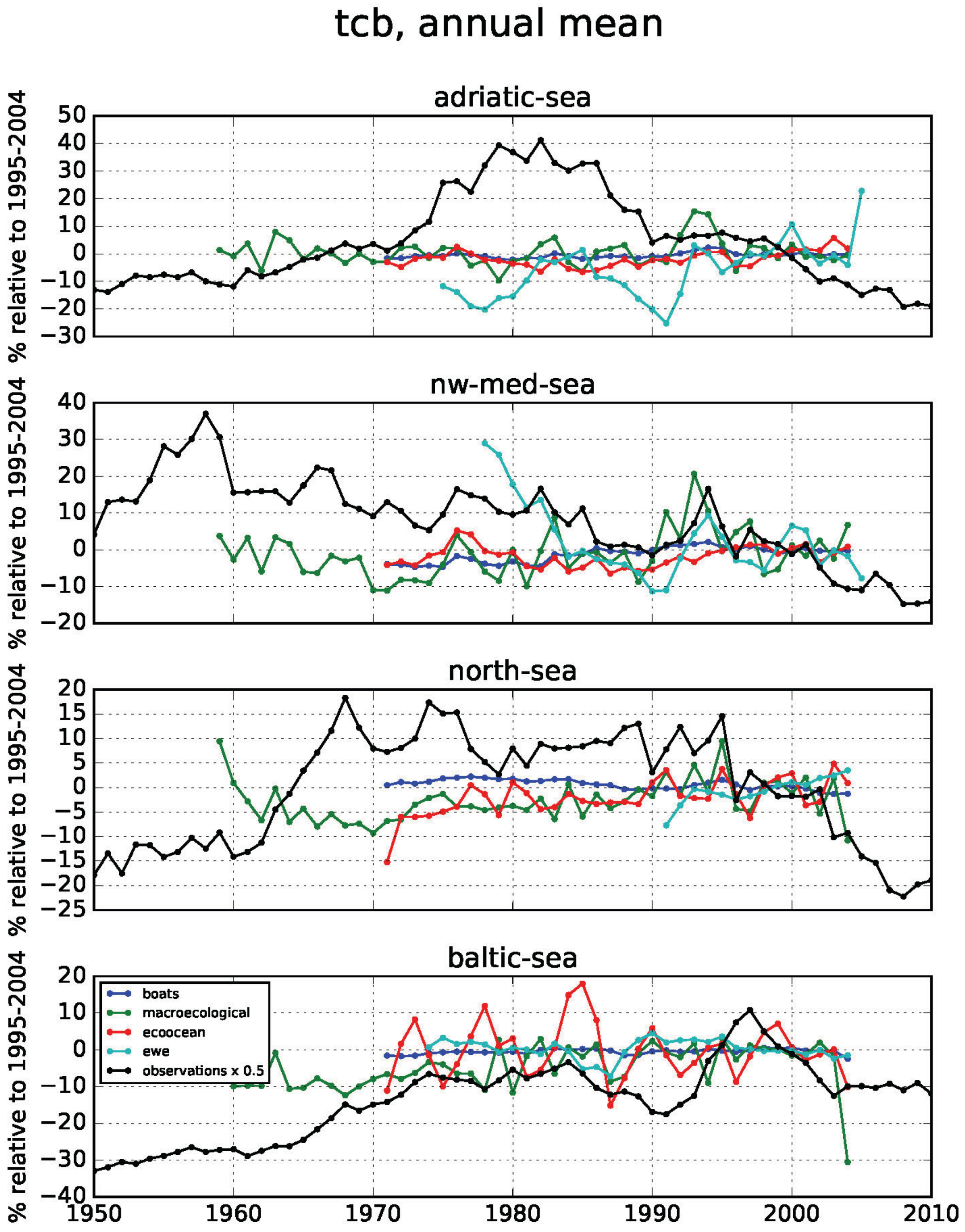

Supplementary Figure 16 Annual mean total consumer biomass (tcb) in three global marine ecosystem models (MACROECOLOGICAL, BOATS, and EcoOcean), one regional model (basin-specific versions of EwE), and observational estimates (Sea Around Us Project (66, 67), black) in four European (sub-)basins. To remove differences in mean levels, deviations from the 1995-2004 average are shown.

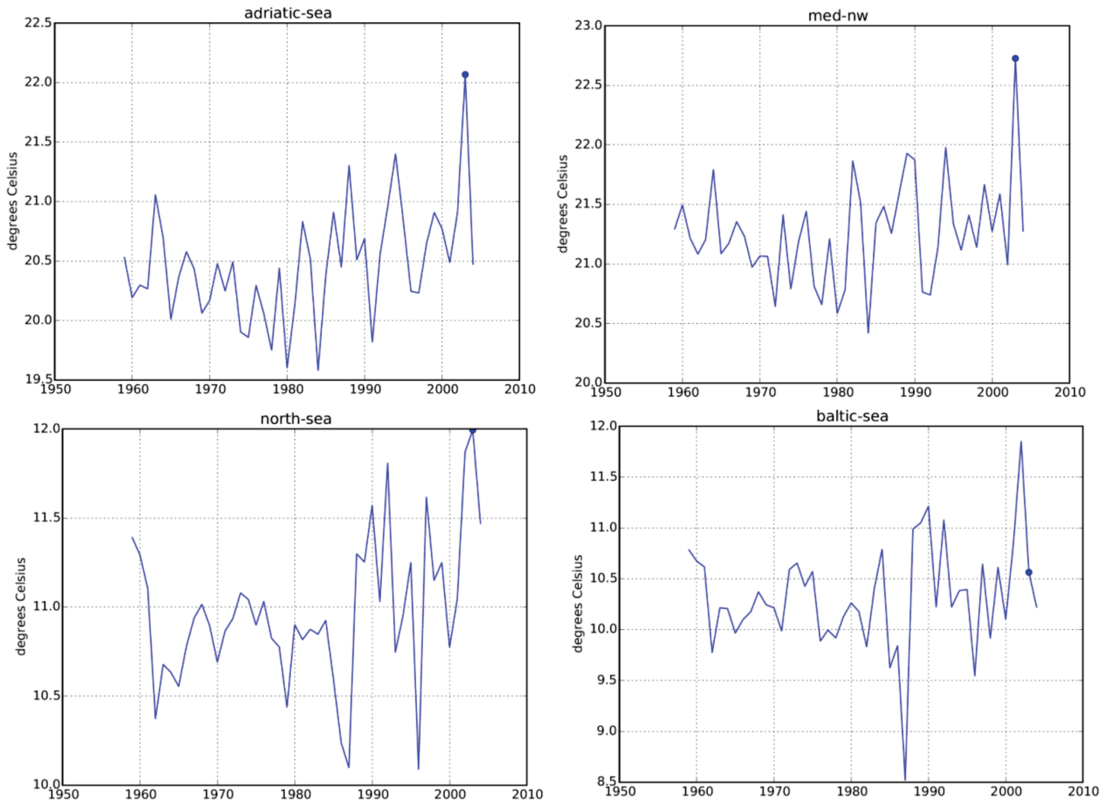

Supplementary Figure 17 Summer (JJA) sea surface temperatures (SST) in the GFDL_reanalysis forcing dataset, for the Adriatic and Northwestern Mediterranean subbasins and the North Sea and Baltic Sea. The year 2003 is marked with a small circle.

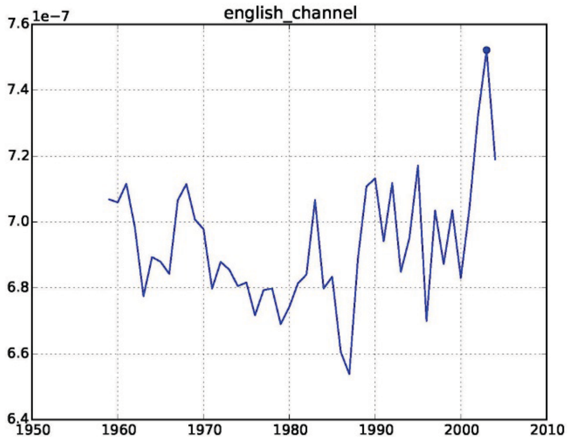

Supplementary Figure 18 Phytoplankton abundance in the English Channel in the GFDL_reanalysis forcing dataset. The year 2003 is marked with a small circle.

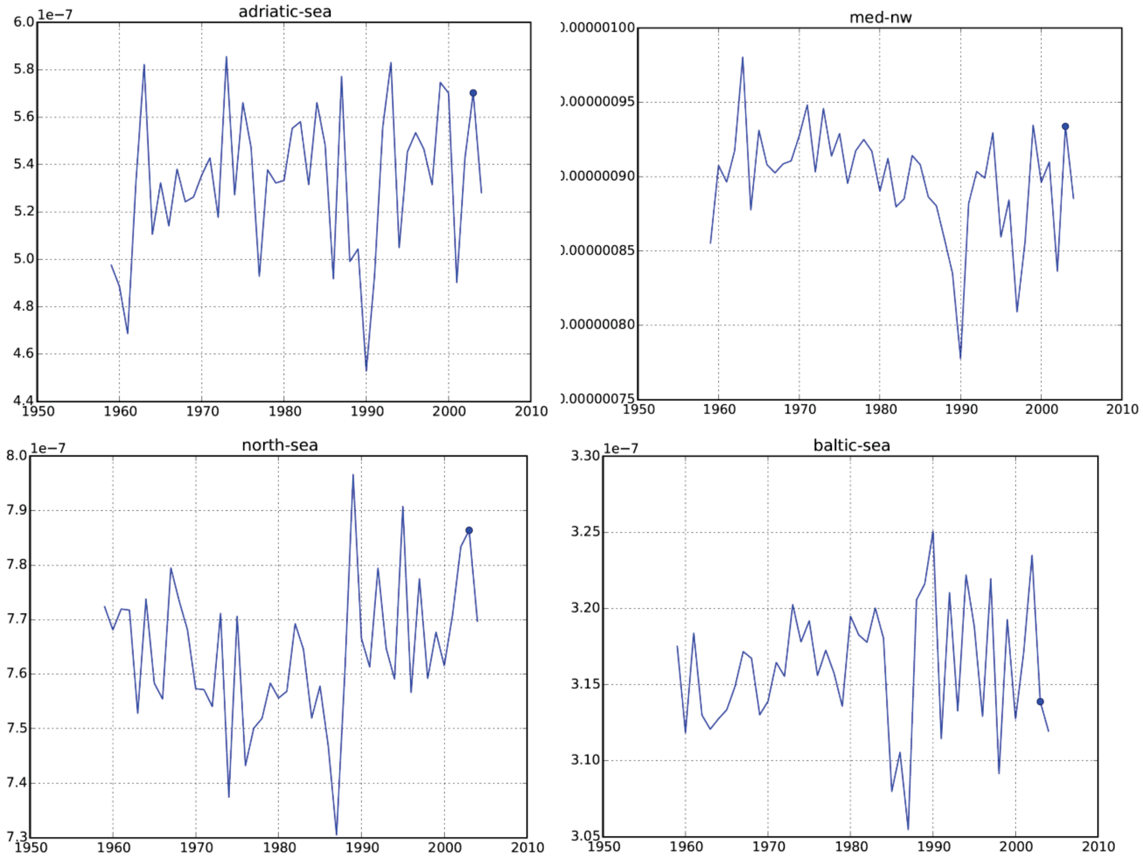

Supplementary Figure 19 Summer (JJA) depth-integrated net primary productivity (NPP) in the GFDL_reanalysis forcing dataset, for the Adriatic and Northwestern Mediterranean subbasins and the North Sea and Baltic Sea. The year 2003 is marked with a small circle.

Methods: Shifted crop yield series, Supplementary Figures 20 – 22

Time-shifts in wheat yields

Depending on whether winter or spring wheat is simulated, sowing and harvest may occur in the same year or in subsequent years; and because different crop models may simulate the one or the other variety, and only report a sequence of harvests, it cannot always be determined whether the first wheat harvest in the 1979-2008 simulation occurred in 1979 or in 1980. Ref. (68) discusses this ambiguity in assigning harvests to calendar years – which is also present in FAOSTAT data – and reports that shifting the country-level simulated yield time series by one year can improve correlation with reported yields for some models and some countries. We also find a few model-country combinations where the time series correlation coefficient improves by more than 0.3 when simulated wheat yields are shifted forwards or backwards by one year. However, applying these time shifts leads only to a marginal improvement of the multi-model median match with the reported yields anomaly in 2003, in Germany, Bulgaria, Romania, Hungary, Poland, Spain, and Austria (Supplementary Figure 22).

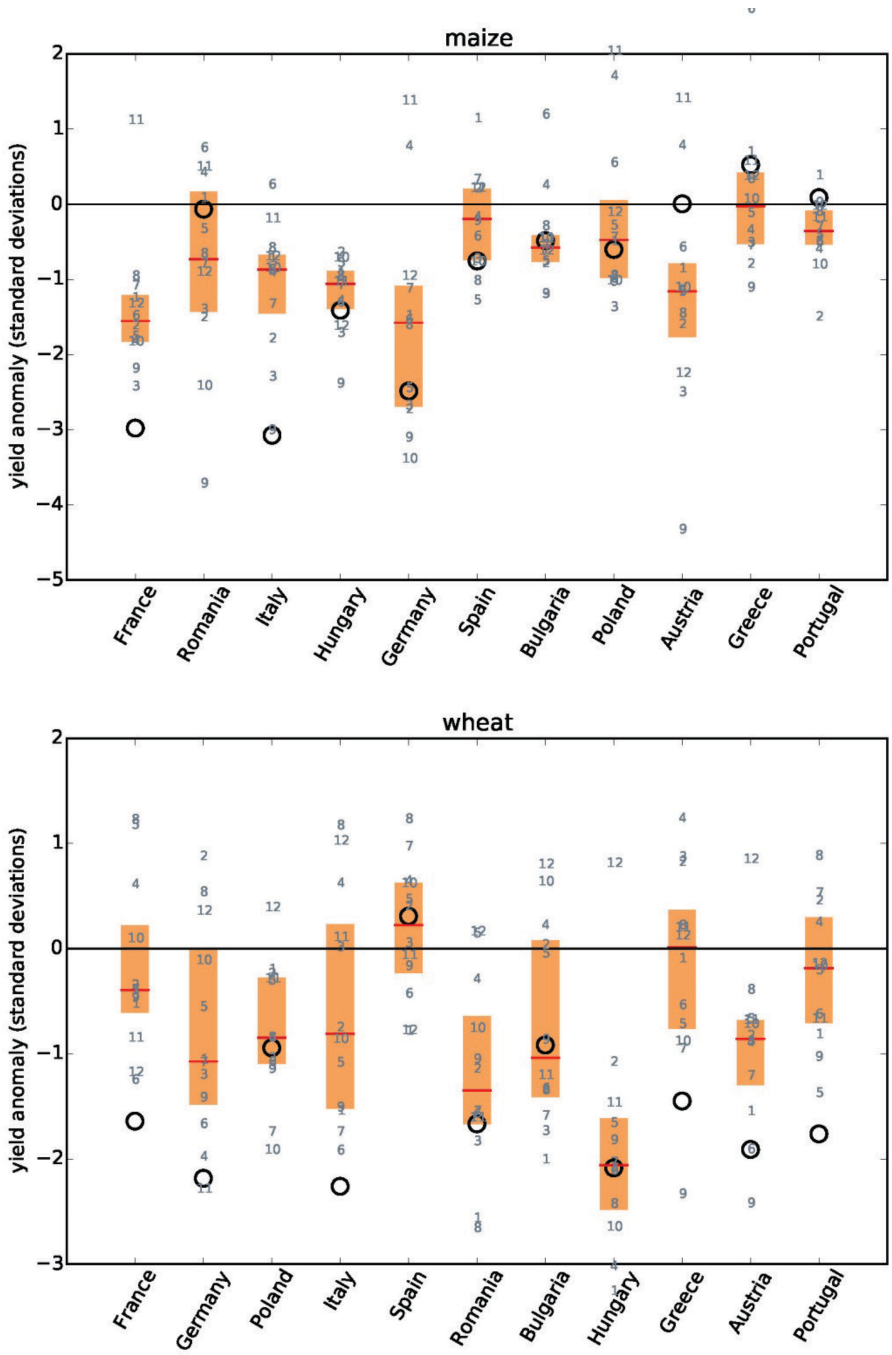

Supplementary Figure 20 As Fig. 3 in the main paper, but with a linear trend removed instead of a moving average.

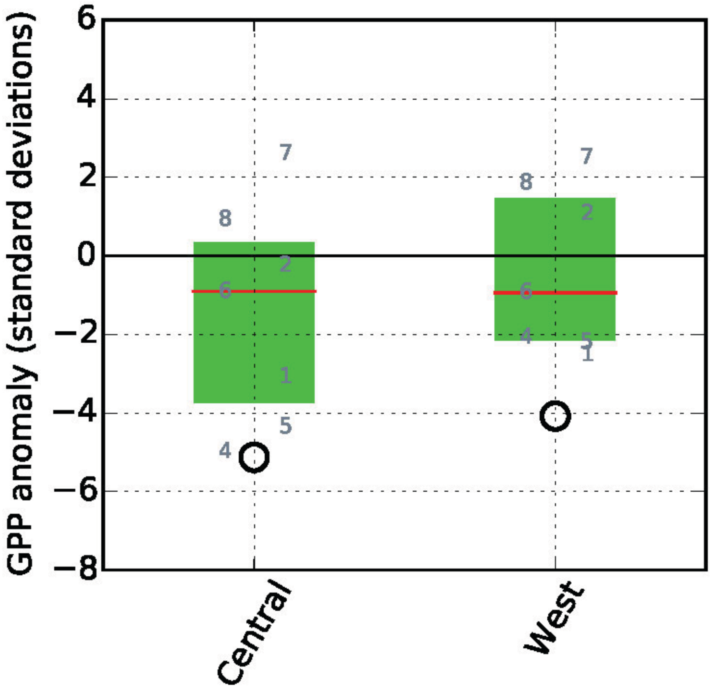

Supplementary Figure 21 As Fig. 4 (a) in the main paper but with the standard deviation of the model GPP anomaly calculated over the entire period 1979-2010, rather than just 2000-2010.

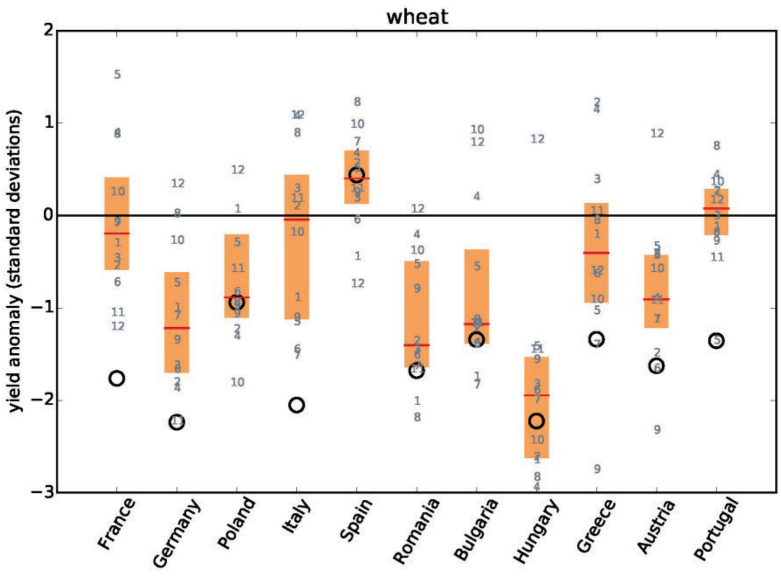

Supplementary Figure 22 As Fig. 3 (b) in the main paper but with the simulated country-level yield time series shifted by one year forwards or backwards in cases where such a shift improves the correlation coefficient between the simulated and the reported yield time series by more than 0.3.

References

1. Weedon GP, et al. (2011) Creation of the WATCH Forcing Data and Its Use to Assess Global and Regional Reference Crop Evaporation over Land during the Twentieth Century. *J Hydrometeorol* 12(5):823–848.

2. Weedon GP, et al. (2014) The WFDEI meteorological forcing data set: WATCH Forcing Data

methodology applied to ERA-Interim reanalysis data. *Water Resour Res* 50(9):7505–7514.

3. van den Hurk B, et al. (2016) LS3MIP (v1.0) contribution to CMIP6: the Land Surface, Snow and Soil moisture Model Intercomparison Project – aims, setup and expected outcome. *Geosci Model Dev* 9(8):2809–2832.

4. Sheffield J, Goteti G, Wood EF (2006) Development of a 50-Year High-Resolution Global Dataset of Meteorological Forcings for Land Surface Modeling. *J Clim* 19(13):3088–3111.

5. Diepen CA, Wolf J, Keulen H, Rappoldt C (1989) WOFOST: a simulation model of crop production. *Soil Use Manag* 5(1):16–24.

6. Boogaard HL, De Wit AJW, te Roller JA, Van Diepen CA (2014) WOFOST Control Centre 2.1; User's Guide for the WOFOST Control Centre 2.1 and the Crop Growth Simulation Model WOFOST 7.1. 7. *Wageningen, The Netherlands: Alterra*.

7. Drewniak B, Song J, Prell J, Kotamarthi VR, Jacob R (2013) Modeling agriculture in the Community Land Model. *Geosci Model Dev* 6(2):495–515.

8. Izaurralde RC, Williams JR, McGill WB, Rosenberg NJ, Jakas MCQ (2006) Simulating soil C dynamics with EPIC: Model description and testing against long-term data. *Ecol Modell* 192(3–4):362–384.

9. Balkovič J, et al. (2014) Global wheat production potentials and management flexibility under the representative concentration pathways. *Glob Planet Change* 122:107–121.

10. Liu J, Williams JR, Zehnder AJB, Yang H (2007) GEPIC - modelling wheat yield and crop water productivity with high resolution on a global scale. *Agric Syst* 94(2):478–493.

11. Folberth C, Gaiser T, Abbaspour KC, Schulin R, Yang H (2012) Regionalization of a large-scale crop growth model for sub-Saharan Africa: Model setup, evaluation, and estimation of maize yields. *Agric Ecosyst Environ* 151:21–33.

12. Lindeskog M, et al. (2013) Implications of accounting for land use in simulations of ecosystem carbon cycling in Africa. *Earth Syst Dyn* 4(2):385–407.

13. BONDEAU A, et al. (2007) Modelling the role of agriculture for the 20th century global terrestrial carbon balance. *Glob Chang Biol* 13(3):679–706.

14. Wu X, et al. (2016) ORCHIDEE-CROP (v0), a new process-based agro-land surface model: model description and evaluation over Europe. *Geosci Model Dev* 9(2):857–873.

15. Elliott J, et al. (2014) The parallel system for integrating impact models and sectors (pSIMS). *Environ Model Softw* 62:509–516.

16. Deryng D, Conway D, Ramankutty N, Price J, Warren R (2014) Global crop yield response to extreme heat stress under multiple climate change futures. *Environ Res Lett* 9(3):034011.

17. Sakurai G, Iizumi T, Nishimori M, Yokozawa M (2015) How much has the increase in atmospheric CO2 directly affected past soybean production? *Sci Rep* 4(1):4978.

18. Dury M, et al. (2011) Responses of European forest ecosystems to 21 st century climate: assessing changes in interannual variability and fire intensity. *iForest - Biogeosciences For* 4(2):82–99.

19. Tian H, et al. (2015) North American terrestrial CO2 uptake largely offset by CH4 and N2O emissions: toward a full accounting of the greenhouse gas budget. *Clim Change* 129(3–

4):413–426.

20. Pan S, et al. (2015) Impacts of climate variability and extremes on global net primary production in the first decade of the 21st century. *J Geogr Sci* 25(9):1027–1044.

21. Harper A, et al. (2016) Improved representation of plant functional types and physiology in the Joint UK Land Environment Simulator (JULES v4.2) using plant trait information. *Geosci Model Dev Discuss*:1–64.

22. Smith B, et al. (2014) Implications of incorporating N cycling and N limitations on primary production in an individual-based dynamic vegetation model. *Biogeosciences* 11(7):2027–2054.

23. Sitch S, et al. (2003) Evaluation of ecosystem dynamics, plant geography and terrestrial carbon cycling in the LPJ dynamic global vegetation model. *Glob Chang Biol* 9(2):161–185.

24. Krinner G (2005) A dynamic global vegetation model for studies of the coupled atmosphere-biosphere system. *Global Biogeochem Cycles* 19(1):GB1015.

25. Chang J, et al. (2017) Benchmarking carbon fluxes of the ISIMIP2a biome models. *Environ Res Lett* 12(4):045002.

26. Zeng N, Mariotti A, Wetzel P (2005) Terrestrial mechanisms of interannual CO 2 variability. *Global Biogeochem Cycles* 19(1). doi:10.1029/2004GB002273.

27. Ito A, Inatomi M (2012) Water-Use Efficiency of the Terrestrial Biosphere: A Model Analysis Focusing on Interactions between the Global Carbon and Water Cycles. *J Hydrometeorol* 13(2):681–694.

28. Tang Q, Oki T, Kanae S, Hu H (2007) The Influence of Precipitation Variability and Partial Irrigation within Grid Cells on a Hydrological Simulation. *J Hydrometeorol* 8(3):499–512.

29. Hanasaki N, et al. (2008) An integrated model for the assessment of global water resources – Part 2: Applications and assessments. *Hydrol Earth Syst Sci* 12(4):1027–1037.

30. Rost S, et al. (2008) Agricultural green and blue water consumption and its influence on the global water system. *Water Resour Res* 44(9):1–17.

31. Pokhrel YN, et al. (2015) Incorporation of groundwater pumping in a global Land Surface Model with the representation of human impacts. *Water Resour Res* 51(1):78–96.

32. Stacke T, Hagemann S (2012) Development and evaluation of a global dynamical wetlands extent scheme. *Hydrol Earth Syst Sci* 16(8):2915–2933.

33. Wada Y, Wisser D, Bierkens MFP (2014) Global modeling of withdrawal, allocation and consumptive use of surface water and groundwater resources. *Earth Syst Dyn* 5(1):15–40.

34. Müller Schmied H, et al. (2016) Variations of global and continental water balance components as impacted by climate forcing uncertainty and human water use. *Hydrol Earth Syst Sci* 20(7):2877–2898.

35. Galbraith ED, Carozza DA, Bianchi D (2017) A coupled human-Earth model perspective on long-term trends in the global marine fishery. *Nat Commun* 8:14884.

36. Christensen V, et al. (2015) The global ocean is an ecosystem: simulating marine life and fisheries. *Glob Ecol Biogeogr* 24(5):507–517.

37. Jennings S, Collingridge K (2015) Predicting Consumer Biomass, Size-Structure, Production, Catch Potential, Responses to Fishing and Associated Uncertainties in the World's Marine Ecosystems. *PLoS One* 10(7):e0133794.

38. Coll M, Santojanni A, Palomera I, Tudela S, Arneri E (2007) An ecological model of the Northern and Central Adriatic Sea: Analysis of ecosystem structure and fishing impacts. *J Mar Syst* 67(1):119–154.

39. Niiranen S, et al. (2013) Combined effects of global climate change and regional ecosystem drivers on an exploited marine food web. *Glob Chang Biol*:n/a-n/a.

40. Christensen V, Walters CJ (2004) Ecopath with Ecosim: methods, capabilities and limitations. *Ecol Modell* 172(2–4):109–139.

41. van Vliet MTH, Wiberg D, Leduc S, Riahi K (2016) Power-generation system vulnerability and adaptation to changes in climate and water resources. *Nat Clim Chang* 6(4):375–380.

42. Baccini M, et al. (2008) Heat Effects on Mortality in 15 European Cities. *Epidemiology* 19(5):711–719.

43. Gosling SN, et al. (2017) Adaptation to Climate Change: A Comparative Analysis of Modeling Methods for Heat-Related Mortality. *Environ Health Perspect* 125(8):1–45.

44. Copa-Cogeca (2004) *Assessment of the impact of the heat wave and drought of the summer 2003 on agriculture and forestry* Available at: http://docs.gip-ecofor.org/libre/COPA_COGECA_2004.pdf.

45. Running SW, Zhao M (2015) Daily GPP and annual NPP (MOD17A2/A3) products NASA Earth Observing System MODIS land algorithm. *MOD17 User's Guid*. Available at: https://www.ntsg.umt.edu/files/modis/MOD17UsersGuide2015_v3.pdf.

46. Reichstein M, et al. (2007) Reduction of ecosystem productivity and respiration during the European summer 2003 climate anomaly: a joint flux tower, remote sensing and modelling analysis. *Glob Chang Biol* 13(3):634–651.

47. Tobin I, et al. (2015) Assessing climate change impacts on European wind energy from ENSEMBLES high-resolution climate projections. *Clim Change* 128(1–2):99–112.

48. Gasparrini A, Leone M (2014) Attributable risk from distributed lag models. *BMC Med Res Methodol* 14(1):55.

49. Mitchell D, et al. (2016) Attributing human mortality during extreme heat waves to anthropogenic climate change. *Environ Res Lett* 11(7):074006.

50. Monteith JL (John L, Unsworth M (2007) *Principles of Environmental Physics.* (Elsevier Science).

51. Borrell C, et al. (2006) Socioeconomic position and excess mortality during the heat wave of 2003 in Barcelona. *Eur J Epidemiol* 21(9):633–640.

52. Michelozzi P, et al. (2005) The impact of the summer 2003 heat waves on mortality in four Italian cities. *Euro Surveill* 10(7):161–5.

53. Michelozzi P, et al. (2006) Temperature and summer mortality: geographical and temporal variations in four Italian cities. *J Epidemiol Community Health* 60:417–423.

54. Grize L, Huss A, Thommen O, Schindler C, Braun-Fahrländer C (2005) Heat wave 2003 and

mortality in Switzerland. *Swiss Med Wkly* 135(13–14):200–205.

55. Martínez Navarro F, Simón-Soria F, López-Abente G (2004) Valoración del impacto de la ola de calor del verano de 2003 sobre la mortalidad. *Gac Sanit* 18(Supl.1):250–258.

56. Tobías A, et al. (2010) Short-term effects of extreme hot summer temperatures on total daily mortality in Barcelona, Spain. *Int J Biometeorol* 54(2):115–117.

57. Le Tertre A, et al. (2006) Impact of the 2003 Heatwave on All-Cause Mortality in 9 French Cities. *Epidemiology* 17(1):75–79.

58. Johnson H, et al. (2005) The impact of the 2003 heat wave on mortality and hospital admissions in England. *Heal Stat Q* (25):6–11.

59. Hajat S, et al. (2006) Impact of High Temperatures on Mortality. *Epidemiology* 17(6):632–638.

60. Gasparrini A, Armstrong B (2011) The Impact of Heat Waves on Mortality. *Epidemiology* 22(1):68–73.

61. Gosling SN, Lowe JA, McGregor GR, Pelling M, Malamud BD (2009) Associations between elevated atmospheric temperature and human mortality: a critical review of the literature. *Clim Change* 92(3–4):299–341.

62. Gosling SN, McGregor GR, Lowe JA (2012) The benefits of quantifying climate model uncertainty in climate change impacts assessment: an example with heat-related mortality change estimates. *Clim Change* 112(2):217–231.

63. Kingsley SL, Eliot MN, Gold J, Vanderslice RR, Wellenius GA (2015) Current and Projected Heat-Related Morbidity and Mortality in Rhode Island. *Environ Health Perspect* 124(4):460–7.

64. Smargiassi A, et al. (2009) Variation of daily warm season mortality as a function of micro-urban heat islands. *J Epidemiol Community Heal* 63(8):659–664.

65. García-Herrera R, Díaz J, Trigo RM, Luterbacher J, Fischer EM (2010) A Review of the European Summer Heat Wave of 2003. *Crit Rev Environ Sci Technol* 40(4):267–306.

66. Pauly D, Zeller D (2016) Catch reconstructions reveal that global marine fisheries catches are higher than reported and declining. *Nat Commun* 7:10244.

67. Pauly D, Zeller D (2015) Catch Reconstruction: concepts, methods and data sources. *Online Publ Sea Around Us (www.seaaroundus.org), Univ Br Columbia*.

68. Müller C, et al. (2017) Global gridded crop model evaluation: benchmarking, skills, deficiencies and implications. *Geosci Model Dev* 10(4):1403–1422.
